# Health system and treatment regimen determinants of delayed first-dose antibiotic administration in hospitalized adults in two public health facilities in Kampala, Uganda

**DOI:** 10.1186/s12913-026-14853-z

**Published:** 2026-06-04

**Authors:** Nsereko Mansoor Kalimunda, Hood Ibanda, Denis Omali, Isaac Magulu Kimbowa, Ronald Kiguba

**Affiliations:** 1 Department of Pharmacology and Therapeutics, Makerere University College of Health Sciences, Kampala, Uganda; 2 Directorate of Research and Grants, Kabale University, Kabale, Uganda; 3 Department of Pharmacology and Therapeutics, Kabale University, Kabale, Uganda; 4 Department of Pharmacology, Faculty of Health Sciences, Islamic University in Uganda, Kampala Campus, Kampala, Uganda

**Keywords:** Antibiotic initiation, Time-to-antibiotics, Health system factors, Antimicrobial stewardship, Hospital inpatients, Length of stay, Uganda

## Abstract

**Background:**

Delayed first-dose antibiotic administration among hospitalized adults partly contributes to poor treatment outcomes. However, the operational drivers of such delays remain poorly characterized in low-resource settings. We sought to delineate the possible operational drivers of these delays, and suggest potential interventions to improve timely antibiotic administration.

**Methods:**

We conducted a prospective cohort study among 606 adult inpatients with suspected or confirmed bacterial infections at two public health facilities in Uganda. Delayed administration was defined as administration of the first antibiotic dose >48 h after prescription. Data were obtained through interviewer-administered questionnaires and medical record review. Data analysis was performed using penalized multivariable logistic regression using Firth’s bias-reduction method to identify factors independently associated with delay.

**Results:**

Among 606 participants, 36 (6%) experienced delayed first-dose antibiotic administration. Participants initiated on three antibiotics had significantly higher odds of delay compared with those prescribed monotherapy (aOR 5.27, 95% CI 1.85–14.97). External medication procurement demonstrated a borderline inverse association with delayed administration (aOR 0.24, 95% CI 0.06–1.03). Sensitivity analysis using a > 24-hour threshold showed directionally consistent findings. In bivariate analyses, participants reporting inadequate household income had lower odds of delayed administration. Generally, patient demographic characteristics were not associated with delay.

**Conclusions:**

Although delayed first-dose antibiotic administration was uncommon, it was driven by modifiable health system inefficiencies and regimen complexity. Treatment regimen complexity was associated with delay, highlighting opportunities to strengthen inpatient antibiotic delivery systems through streamlined multi-drug prescribing and administration workflows. Larger multicenter studies are warranted to assess the clinical implications of delayed antibiotic administration in resource-limited settings.

## Introduction

The first-dose of an antibiotic is a critical intervention in the management of acute bacterial infections [1]. The goal is not just to give antibiotics, but to give the right antibiotic as soon as possible after the need is identified. In conditions like sepsis, severe pneumonia, and meningitis, every hour of delay in antibiotic administration is associated with a significant increase in mortality and morbidity [2–4]. The principle of “timeliness” in first-dose antibiotic administration therefore remains cornerstone to effective antimicrobial therapy as guided by international guidelines for sepsis and severe infection [1, 5]. However, in low- and middle-income countries (LMICs), health system constraints often make timely administration a major challenge [6, 7].

For example, a study on pneumonia patients in Malawian referral hospital found that bottlenecks/challenges in triage and consultation were primary drivers of delays in administering the first antibiotic dose [8]. Similarly, a research study conducted in Kisantu district hospital in Democratic Republic of Congo reported that 38.2% (*n* = 703/1838) of the admitted children had their antibiotics not administered on the day of prescription. The study further attributed delayed first-dose antibiotic administration to factors such as; inability to afford the medicines, antibiotic stockouts, inaccessibility and nursing staff failure to administer the antibiotic [9]. These system-level failures including drug stock-outs, understaffing and inefficient patient flow are compounded by patient-related bottlenecks like financial constraints and delays in seeking care, creating a perfect storm for suboptimal treatment initiation [6, 10].

Consequently, delayed antibiotic first-dose administration is likely associated with prolonged infection and clinical deterioration, and hence longer hospital stays, increased healthcare costs, and a greater burden on already strained systems [11]. Furthermore, delayed or inadequate initial therapy is a recognized driver of antimicrobial resistance (AMR) [10, 12]. When treatment is delayed, therapeutic drug concentrations are hardly attained. This is partly due to potential time-dependent increase in bacterial load. This cascade of events (delayed first-dose antibiotic administration-increased bacterial load-subtherapeutic drug concentrations) potentially contributes to AMR, which complicates antimicrobial therapy and worsens treatment outcomes [10, 13].

In Uganda, while some studies have documented high rates of antibiotic prescription, the focus has rarely been on the timeliness of administration after the clinical decision is made [14]. Existing research has highlighted the limitation of financial barriers and prescribing patterns but lacks a granular analysis of the specific intra-hospital bottlenecks that cause delays after a patient has been admitted with a written prescription [14]. However, previous studies did not clearly describe the association of selected factors with delays in administering the first-dose of antibiotics in adult inpatients, which warranted the current study. Using a three-domain framework, we sought to determine whether modifiable patient-, treatment-, and health system-related factors are associated with delayed antibiotic administration in adult inpatients.

The association between delayed first-dose antibiotic administration and patient-related factors such as age, sex, education level and self-reported household income status was determined. We further assessed whether treatment-related and health system-related factors such as treatment regimen complexity and time to initial consultation respectively, were associated with delay in antibiotic administration. Findings reported here could support targeted interventions, such as antimicrobial stewardship programs and operational improvements that are designed to optimize patient outcomes in Uganda and similar resource-constrained settings.

## Methods

### Study aim

The current study aimed to identify health system and treatment regimen determinants of delayed first-dose antibiotic administration in adult inpatients in Uganda.

### Study design

We conducted a prospective cohort study from April 2023 to June 2023 among adult patients admitted with suspected or confirmed bacterial infections at Kiruddu National Referral Hospital and Kisenyi Health Centre IV in Kampala, Uganda. Consenting adult inpatients were enrolled, and their time of admission to the time of first-dose antibiotic administration were noted.

### Study setting

Uganda’s health system follows an integrated structure comprising approximately 6,937 health facilities, where 45% are public-owned, 40% are private for-profit (PFP), and 15% are private-not-for-profit (PNFPs) [15]. Briefly, Kiruddu National Referral Hospital offers tertiary care with about 200 inpatient beds and Kisenyi Health Centre IV offers secondary care with about 50 inpatient beds. These sites were selected to represent different levels of Uganda’s hierarchical healthcare system [15].

### Characteristics of study participants

#### Exclusion criteria

Critically ill patients were excluded because they often receive specialized emergency care and rapid antibiotic administration pathways that differ from routine inpatient care. Patients without documented medical history and those receiving topical and prophylactic antibiotics were excluded. Patients that received antibiotics prior to admission to study sites were also excluded, since this could be associated with in pill burden and “prescription hesitancy”, both of which influence adherence.

#### Inclusion criteria

Adult patients aged 18 years or older and hospitalized with suspected or confirmed bacterial infections based on clinical or laboratory diagnosis were enrolled. All participants received systemic antibiotics during admission.

### Sample size determination

We determined the study sample size using the Kish Leslie formula for a single population proportion: N = (Zα² * P * (1-P)) / δ². We used a conservative prevalence (P) of 50% for delayed first-dose antibiotic administration in adult in-patients to maximize the sample size. With z_α_ = 1.96 (95% confidence level) and δ = 0.05 (5% margin of error), the calculation yielded a minimum of 384 participants. After adding 10% for non-response, the target was 422. However, as we employed a two-stage sampling strategy involving proportionate sampling from two facilities of different sizes, we anticipated a design effect. A design effect of 1.5 was applied to adjust for the loss of efficiency compared to simple random sampling. The adjusted sample size was calculated as 422 * 1.5 = 633.

Finally, to achieve logical allocation from the two study sites based on average monthly admission rates over the preceding 12 months (Kiruddu NRH: 3000; Kisenyi HCIV: 600) regardless of disease cause, the sample proportioned. To ensure a practical and implementable sampling interval at both sites, the sample size was adjusted to 606, which allowed for a consistent sampling interval of 6 at both facilities while maintaining a strong representation of the source population as presented below.

Kisenyi HCIV: (600 / 3600) * 606 = 101.

Kiruddu NRH: (3000 / 3600) * 606 = 505.

### Sampling procedure

A two-stage sampling approach was employed. In the first stage, two public health facilities Kiruddu National Referral Hospital and Kisenyi Health Centre IV were purposively selected to represent different levels of care within Uganda’s public health system. In the second stage, a systematic sampling method was used to recruit individual participants within each facility. The sample size was allocated to each facility using probability proportionate to size (PPS), based on their average monthly admission rates over the preceding 12 months (Kiruddu NRH: 3000; Kisenyi HCIV: 600). This PPS calculation determined that 505 participants would be recruited from Kiruddu NRH and 101 from Kisenyi HCIV, ensuring the sample was representative of the patient volume at each site. The sampling interval was then calculated separately for each facility by dividing the estimated number of eligible patients by the required sample size, resulting in an interval of 6 at both sites. The first participant on each ward was selected using simple random sampling (by picking a number between 1 and 6), after which every 6th eligible patient was invited to participate. If a selected patient was ineligible, the next consecutive eligible patient was enrolled.

### Data collection tools

Data were collected using an interviewer-administered questionnaire adapted, with permission and minor modifications, from a previous related study [14]. The questionnaire’s face and content validity were rigorously assessed by a multidisciplinary team comprising a pharmacologist, pharmacist, epidemiologist and public health specialist. The tool was subsequently piloted on 10 adult in-patients at Kisenyi Health Centre IV. Feedback regarding question wording and clarity was incorporated into the final instrument, and data from this pilot phase were excluded from the final analysis. The final questionnaire comprised four comprehensive sections;

Section A: Facility Identifiers, Screening, and Consent.

Section B: Socio-demographic Characteristics.

Section C: Clinical and Temporal Data.

Section D: Health System Factors.

### Variables and definitions

The primary outcome of this study was delayed first-dose antibiotic administration. This was defined as the administration of the first antibiotic dose > 48 h after the time of prescription. The outcome was measured using data extracted from patient medical records, which documented the precise date and time of the antibiotic prescription and the corresponding date and time of the first administered dose.

Independent variables investigated for their association with antibiotic delay were categorized into three groups: Patient-related factors: Age, sex, marital status, place of residence, education level, and self-reported household income status. Treatment-related factors: The complexity of the initial antibiotic regimen (categorized as 1, 2, or ≥ 3 antibiotics) and the primary disease category. Health system-related factors: Time to initial consultation (> 12 h versus ≤ 12 h), facility accessibility, lack of convenient facility hours, inadequate staffing, and the need for external antibiotic procurement.

### Data collection and management

Study data were collected using a validated interviewer-administered questionnaire, and supplemented by medical record reviews. Four trained research assistants (three at Kiruddu NRH, one at Kisenyi HCIV) screened patients for eligibility, obtained written informed consent at admission, and administered the questionnaires (the entire processing taking about 15 min). Research assistants reviewed patients’ medical files to extract clinical and temporal data. Completed questionnaires were stored in a lockable safety cabinet and collected weekly for review by the Principal Investigator. Data collection was finalized and verified against medical records at discharge or death.

Daily data review was performed to identify and correct any missing variables. During data cleaning, files with major missing variables were removed from the analysis. Data were double-entered and validated using EpiData version 4.4.2.1 before export to R statistical software version 4.x (R Foundation for Statistical Computing, Vienna, Austria) for cleaning and analysis.

### Statistical data analysis

#### Data presentation

Data were cleaned and analyzed using R statistical software version 4.x (R Foundation for Statistical Computing, Vienna, Austria). The primary study outcome was delayed first-dose antibiotic administration, defined as receipt of the first prescribed antibiotic dose > 48 h after prescription. A sensitivity analysis using a > 24-hour threshold was conducted to evaluate the robustness of observed associations under a less severe delay. Descriptive statistics were used to summarize participant characteristics and health system variables. Categorical variables were summarized using frequencies and percentages. Continuous variables were summarized using medians and interquartile ranges (IQRs) because delay durations were right-skewed. Means and ranges were additionally reported to describe variability in delay times.

### Logistic regression analysis

Bivariate logistic regression analyses were performed to evaluate unadjusted associations between candidate explanatory variables and delayed antibiotic administration > 48 h. Crude odds ratios (cORs), 95% confidence intervals (CIs) and corresponding p-values were estimated. Variables with *p* < 0.20 during bivariate analysis were considered for multivariable modeling. Additionally, variables with established clinical or health-system relevance were considered for inclusion irrespective of bivariate statistical significance to minimize residual confounding. All statistical tests were two-sided, and statistical significance was assessed at an alpha level of 0.05.

Because only 36 severe delay events (> 48 h) were identified, the events-per-variable ratio for conventional logistic regression was below the commonly recommended threshold for stable maximum likelihood estimation. Consequently, penalized multivariable logistic regression using Firth’s bias-reduction method was performed to minimize sparse-data bias, reduce small-sample instability, and mitigate risks of model overfitting. Adjusted odds ratios (aORs), 95% confidence intervals, and p-values were estimated from the penalized regression. Sensitivity analyses were conducted using a delay of > 24 h to evaluate the consistency of observed associations under a less restrictive operational definition of delay. Multicollinearity among explanatory variables included in the multivariable model was assessed using variance inflation factors (VIFs). The VIF values below 5 indicated acceptable collinearity levels. Model calibration and goodness-of-fit were additionally to evaluate overall model adequacy.

## Results

### Demographics of study participants

A total of 606 adult inpatients prescribed antibiotics were included in the final analysis. Delayed first-dose antibiotic administration > 48 h after prescription occurred in 36 participants (5.9%). Using the secondary > 24-hour threshold, 294 participants (48.5%) experienced delayed antibiotic administration. The median time from antibiotic prescription to administration was 23.3 h (IQR: 11.4–37.4 h), while the mean delay was 28.9 h (range: 0.1–196.9 h). Most participants were recruited from Kiruddu National Referral Hospital (83.3%), and females constituted the majority of participants. Gastrointestinal, obstetric/gynecological, respiratory, and cardiovascular conditions were the most common disease categories (Table 1).

**Table 1:** Baseline socio-demographic and clinical characteristics of study participants

Characteristic	No delay *N* = 570^1^	Delay *N* = 36^1^	*p*-value^2^
*Health facility*			0.2
Kiruddu NRH	478 (84%)	27 (75%)	
Kisenyi HCIV	92 (16%)	9 (25%)	
*Sex*			0.2
Female	380 (67%)	20 (56%)	
Male	190 (33%)	16 (44%)	
*Age group*			0.3
18–24	132 (23%)	5 (14%)	
25–34	162 (28%)	15 (42%)	
35–44	88 (15%)	6 (17%)	
45+	188 (33%)	10 (28%)	
*Marital status*			0.2
Divorced/Separated/Widowed	124 (22%)	3 (8.3%)	
Married	349 (61%)	26 (72%)	
Single	97 (17%)	7 (19%)	
*Participant residence*			0.7
Rural	174 (31%)	10 (28%)	
Urban/suburban	396 (69%)	26 (72%)	
*Education level*			0.4
Advanced college	52 (9.1%)	1 (2.8%)	
No formal education	98 (17%)	8 (22%)	
Primary	160 (28%)	13 (36%)	
Secondary	260 (46%)	14 (39%)	
*Household income status*			**0.001**
Adequate	175 (31%)	22 (61%)	
Inadequate	384 (67%)	14 (39%)	
Surplus	11 (1.9%)	0 (0%)	
*Disease category*			0.4
Cardiovascular	71 (12%)	2 (5.6%)	
Gastrointestinal	156 (27%)	11 (31%)	
Genitourinary	60 (11%)	5 (14%)	
Obstetrics/Gynecology	141 (25%)	10 (28%)	
Other diseases	15 (2.6%)	2 (5.6%)	
Respiratory	95 (17%)	3 (8.3%)	
Skin	32 (5.6%)	3 (8.3%)	
*Initial quantity of antibiotic regimen*			**0.004**
1 antibiotic	237 (42%)	6 (17%)	
2 antibiotics	258 (45%)	20 (56%)	
≥ 3 antibiotics	75 (13%)	10 (28%)	
*Convenient staff working hours*	465 (82%)	24 (67%)	**0.028**
*Adequate staffing*	258 (45%)	15 (42%)	0.7
*External medicines procurement*	111 (19%)	2 (5.6%)	**0.038**
*Time to medical consultation*			0.067
> 12 h	66 (12%)	8 (22%)	
< 12 h	504 (88%)	28 (78%)	

^1^n (%); ^2^Pearson’s Chi-squared test; Fisher’s exact test; N = number of participants

### Bivariate analysis

At bivariate logistic regression analysis, several factors demonstrated associations with delayed first-dose antibiotic administration > 48 h (Table 2). Participants prescribed two antibiotics had higher odds of delayed administration compared with those prescribed monotherapy (cOR 3.06, 95% CI 1.21–7.75, *p* = 0.018). Similarly, participants prescribed three antibiotics had increased odds of delay (cOR 5.27, 95% CI 1.85–14.97, *p* = 0.002). Participants reporting inadequate household income had lower odds of delayed administration than those reporting adequate income (cOR 0.29, 95% CI 0.14–0.58, *p* < 0.001). External medication procurement was also associated with lower odds of delayed administration (cOR 0.24, 95% CI 0.06–1.03, *p* = 0.055), although the association did not reach conventional statistical significance. Variables including age group, marital status, convenient facility hours, and time to medical consultation demonstrated potential clinical relevance and were considered for multivariable analysis based on pre-specified selection criteria.

**Table 2:** Bivariate logistic regression analysis of factors associated with delayed antibiotic administration > 48 h

Variable	Category	*n* (%) delayed	cOR (95% CI)	*P*-value
*Age group*	18–24	5/137 (3.6%)	Reference	
	25–34	15/177 (8.5%)	2.44 (0.87–6.90)	0.091
	35–44	6/94 (6.4%)	1.80 (0.53–6.08)	0.344
	45+	10/198 (5.1%)	1.40 (0.47–4.20)	0.544
*Marital status*	Divorced/ Separated/ Widowed	3/127 (2.4%)	Reference	
	Married	26/375 (6.9%)	3.08 (0.92–10.35)	0.069
	Single	7/104 (6.7%)	2.98 (0.75–11.84)	0.120
*Household income*	Adequate	22/197 (11.2%)	Reference	
	Inadequate	14/398 (3.5%)	0.29 (0.14–0.58)	**0.001**
*Initial antibiotic Regimen*	1 antibiotic	6/243 (2.5%)	Reference	
	2 antibiotics	20/278 (7.2%)	3.06 (1.21–7.75)	**0.018**
	≥ 3 antibiotics	10/85 (11.8%)	5.27 (1.85–14.97)	**0.002**
*Convenient hours*	No	12/117 (10.3%)	Reference	
	Yes	24/489 (4.9%)	0.45 (0.22–0.93)	**0.032**
*Adequate staffing*	No	21/333 (6.3%)	Reference	
	Yes	15/273 (5.5%)	0.86 (0.44–1.71)	0.674
*Time to consultation*	> 12 h	8/74 (10.8%)	Reference	
	< 12 h	28/532 (5.3%)	0.46 (0.20–1.05)	0.064
*External medicines procurement*	No	34/493 (6.9%)	Reference	
	Yes	2/113 (1.8%)	0.24 (0.06–1.03)	0.055

cOR = Crude Odds Ratio; CI = Confidence Interval. Reference categories were automatically selected as baseline comparator groups for categorical logistic regression analyses. Outcome variable was delayed antibiotic administration > 48 h. Variables with *p* < 0.20 and those considered clinically relevant were eligible for multivariable analysis

### Multivariable analysis

Penalized multivariable logistic regression identified independent associations with delayed first-dose antibiotic administration > 48 h (Table 3). Participants prescribed two antibiotics had higher odds of delayed administration compared with those prescribed monotherapy (aOR 2.06, 95% CI 1.33–8.87, *p* = 0.011). Similarly, prescription of three antibiotics was associated with increased odds of delay (aOR 3.14, 95% CI 1.97–16.84, *p* = 0.001). Married participants had higher adjusted odds of delayed administration compared with divorced, separated, or widowed participants (aOR 1.52, 95% CI 1.04–12.50, *p* = 0.043) but with a wide confidence interval. External medication procurement demonstrated a borderline inverse association with delayed administration (aOR 0.37, 95% CI 0.06–1.06, *p* = 0.061).

**Table 3:** Penalized multivariable logistic regression analysis of factors associated with delayed antibiotic administration > 48 h and > 24 h

Variable	aOR > 48 h	95% CI	*p*-value		aOR > 24 h	95% CI	*p*-value	
*Age group: 25–34*	1.7	(0.97–8.20)		0.056	1.0	(0.64–1.58)		0.982
* 35–44*	1.0	(0.47–5.59)		0.448	1.03	(0.65–1.88)		0.719
* 45+*	1.0	(0.39–3.69)		0.744	1.0	(0.64–1.56)		0.994
*Marital status (married)*	1.52	(1.04–12.50)		0.043	1.4	(1.03–2.37)		0.035
*Marital status (single)*	1.27	(0.80-13.46)		0.1	1.02	(0.69–1.99)		0.568
*2 antibiotics*	2.06	(1.33–8.87)		0.011	1.0	(0.70–1.42)		0.998
*≥ 3 antibiotics*	3.14	(1.97–16.84)		0.001	0.93	(0.53–1.46)		0.63
*Adequate staffing (Yes)*	0.9	(0.39–1.62)		0.523	1.0	(0.71–1.37)		0.951
*External medicines procurement (Yes)*	0.37	(0.06–1.06)		0.061	0.6	(0.38–0.89)		0.012

Abbreviations: aOR = Adjusted Odds Ratio; CI = Confidence Interval. Penalized multivariable logistic regression was used to address sparse-data bias, low event frequency, and instability associated with ordinary logistic regression. Variables included in the model were selected based on clinical relevance and bivariate screening criteria (*p* < 0.20)

Sensitivity analysis using > 24-hour delay demonstrated generally consistent findings. Married participants remained at increased odds of delayed administration (aOR 1.40, 95% CI 1.03–2.37, *p* = 0.035), while external medication procurement was significantly associated with lower odds of delay (aOR 0.60, 95% CI 0.38–0.89, *p* = 0.012). In contrast, associations between multiple-antibiotic regimens and delayed administration were not observed under the > 24-hour definition.

### Multicollinearity assessment of explanatory variables

Multicollinearity assessment demonstrated no evidence of problematic collinearity among explanatory variables included in the multivariable model. All VIFs were substantially below the pre-specified threshold of 5, with observed VIF values ranging from 1.02 to 1.68 (Table 4).

**Table 4:** Multicollinearity assessment of explanatory variables included in the multivariable regression analysis

Variable	VIF
Age group (25–34 years)	1.64
Age group (35–44 years)	1.44
Age group (45 + years)	1.68
Married marital status	1.54
Single marital status	1.53
Two-antibiotic regimen	1.18
Three-antibiotic regimen	1.18
Adequate staffing	1.02
External medication procurement	1.03

VIF = Variance Inflation Factor; VIF values < 5 indicated non-multicollinearity

## Discussion

The present study provided evidence from a resource-limited setting where data on first-dose antibiotic administration timing remain scarce. The study investigated determinants of delayed first-dose antibiotic administration among hospitalized adults in two public health facilities in Kampala, Uganda. Delayed administration > 48 h after prescription occurred in a relatively small but clinically important proportion of participants (5.9%). In adjusted analyses, treatment regimen complexity, marital status, and external medication procurement pathways were associated with delayed first-dose antibiotic administration. Furthermore, diagnostics of the regression model demonstrated low multicollinearity among explanatory variables. This indicated that observed associations were unlikely to be substantially influenced by intercorrelation among health-system factors.

The observed low prevalence of delayed first-dose antibiotic administration > 48 h may suggest that most patients received antibiotics within an acceptable timeframe. However, nearly half of the participants 294 (48.5%) experienced delays > 24 h. This indicated inefficiencies in antibiotic delivery systems. Timely antibiotic administration remains essential for optimizing outcomes among patients with bacterial infections where treatment delays are associated with worse clinical outcomes and increased mortality [11, 13].

Participants prescribed three-antibiotic regimens experienced significantly higher odds of delayed administration compared with monotherapy (aOR 3.14, 95% CI 1.97–16.84, *p* = 0.001). This finding suggests that increasing regimen complexity may introduce operational barriers to timely antibiotic administration. Examples of such operational barriers could include medicine availability constraints, delays in prescription processing, increased nursing workload and difficulties coordinating simultaneous administration of multiple medicines. Furthermore, logistical challenges including time required to procure, prepare and administer multiple medications introduce a significant barrier to timely administration [8, 16]. Similar observations were reported in studies examining inpatient medication delays in resource-constrained settings, where regimen complexity contributed to missed or delayed doses due to logistical and stock-related challenges [8, 9]. Furthermore, studies on inpatient antimicrobial stewardship have shown that complex multidrug regimens often require additional verification, procurement, or scheduling processes that may inadvertently delay treatment initiation [17, 18].

The burden of these complex regimens is further illuminated by their specific composition; our data reveal that triple-therapy was distinctly characterized by a heavy reliance on antibiotics from the ‘Other’ category such as glycopeptides (e.g., vancomycin) which constituted over a quarter (27.1%) of all prescriptions in three antibiotics regimens. These agents are typically reserved for severe, multidrug-resistant, or nosocomial infections and often require special handling, or specific monitoring [10, 19]. Furthermore, such reserve antibiotics are frequently less readily available in hospital formularies or require special approval processes, creating procurement bottlenecks that can critically impact time-to-therapy [11, 20]. Therefore, the delay associated with complex regimens is likely not just a function of the number of drugs, but also a consequence of the specific, logistically complex agents that define these advanced treatment protocols.

The observed association between marital status and delayed antibiotic administration should be interpreted cautiously because of the relatively wide confidence intervals. Nonetheless, marital or family structure may influence treatment pathways through differences in caregiver support, decision-making dynamics, financial prioritization or ability to mobilize resources for medicine procurement during hospitalization. Although evidence directly linking marital status to delayed inpatient antibiotic administration remains limited, studies in healthcare utilization reported that social and household dynamics influence healthcare access and treatment adherence in hospitalized populations [21]. Additional studies are needed to better understand this relationship within Ugandan hospital settings.

External medication procurement demonstrated a borderline inverse association with delayed first-dose antibiotic administration in the primary analysis. This finding remained significant in sensitivity analysis at > 24 h. This could be due to the fact that patients who rapidly obtained medicines from external pharmacies were able to circumvent institutional supply bottlenecks. Similar challenges involving medicine stockouts and patient-led procurement were reported in Uganda and Democratic Republic of Congo [9, 14]. However, because external procurement introduces inequities related to affordability and access, reliance on out-of-pocket purchasing may not represent a sustainable strategy for timely antibiotic delivery.

A counterintuitive yet important finding was that patients that reported inadequate household income had lower odds of delayed administration than those reporting adequate income (cOR 0.29, 95% CI 0.14–0.58, *p* < 0.001). This may be explained by the protective effect of Uganda’s tax-funded public health system, which provides free services and may buffer financial barriers for the poorest patients [15]. An alternative explanation is potential clinical prioritization, where healthcare providers may unconsciously perceive poorer patients as being at higher acuity or having fewer alternative care options, leading to expedited processes for them once within the system. This paradoxical finding warrants further qualitative investigation to understand the roles of provider perception and decision-making in adherence to pharmacological therapy, especially timely first-dose antibiotic initiation.

Our findings point to several actionable areas for intervention. To mitigate the risk associated with complex regimens, clinical interventions could include developing standardized protocols and pre-packaged order sets for common multi-drug regimens to streamline their preparation and administration. These multi-faceted strategies are directly aligned with the goals of Uganda’s National Action Plan on antimicrobial resistance [7] and are essential for strengthening health systems and improving patient outcomes in resource-constrained settings, as called for in recent Ugandan scoping reviews on stewardship [22]. Overall, these findings highlight the importance of strengthening operational systems for inpatient antibiotic delivery, particularly for patients prescribed multidrug regimens. Future multicentre studies should investigate institutional and workflow-related barriers to timely antibiotic administration. This will support efforts in evaluating interventions that improve medicine availability and administration efficiency in resource-limited hospitals.

### Limitations of the study

Despite the critical relevance of our findings to public health practice, the current study had some limitations, some of which were inevitable and out of the study context. First, the current study did not report microbiological culture results for participants that were identified as having bacterial infection. This was partly driven by the common practice of empirical treatment in the Ugandan setting, yet mistreatment could directly impact on length of stay in the hospital. Additionally, the study population was predominantly derived from a single tertiary referral hospital, which may limit generalizability to smaller healthcare facilities or lower-level hospitals in Uganda.

Secondly, the observational study design precludes causal inference. Consequently, identified associations should not be interpreted as evidence of causality. Future studies should consider a longer and more robust longitudinal design to determine the possible associated treatment outcomes. Future research studies should also integrate bacterial culture and sensitivity to support the determination of the best antibiotic medicine for a specific bacterial strain. Such a study could draw attention to particular microbes and antibiotic pairs that require maximum attention regarding empirical drug use and urgent administration of antibiotics in public health facilities in LMICs. Additionally, time of admission, pharmacy dispensing time, ward type (surgical vs. medical vs. obstetric), prescriber cadre and antibiotic availability on-ward versus requiring external procurement. Yet such factors could potentially influence the duration within which the first-dose of antibiotics should be administered.

Finally, the current study excluded critically ill patients that would interest policy makers in designing supportive first-dose antibiotic administration guidelines in the local context. This further complicates direct inter-study comparisons and similar studies should be context-specific to elucidate particular challenges specific to the study setting. Therefore, critically ill patients were excluded from the study given their limited access and specialized treatment mostly at tertiary facilities as compared to lower level public health facilities. A similar study should be conducted for critically ill patients in Uganda to support regulatory organs that aim at improving antibiotic stewardship to minimize adverse outcomes and events that are associated with such delayed administration in critically ill patients.

## Conclusions

Severe delayed antibiotic administration > 48 h occurred in approximately 6% of hospitalized adult patients. Surprisingly, nearly half experienced delays > 24 h after antibiotic prescription. Treatment regimen complexity, particularly administration of multiple antibiotics, was associated with increased odds of delayed administration. However, external medication procurement demonstrated an inverse association in sensitivity analyses. These findings suggest that operational barriers surrounding multidrug prescribing and medicine access may contribute to delayed first-dose antibiotic administration in resource-limited hospital settings. Strengthening inpatient antibiotic supply systems, streamlining workflows for multidrug antibiotic administration and improving institutional medicine availability may help optimize timely antibiotic delivery. Further multicentre studies are warranted to better characterize operational determinants of delayed first-dose antibiotic administration and evaluate interventions to improve antibiotic timeliness in low-resource settings.

## Data Availability

All data generated or analysed during this study are included in this published article. Any other data that could be of interest to the reader can be accessible by contacting the corresponding author on reasonable request.

## Abbreviations

AMR: Antimicrobial resistance
aOR: Adjusted odds ratio
CI: Confidence interval
HCIV: Health center IV
LMICs: Low-and middle-income countries
NRH: National referral hospital
OR: Odds ratio
PPS: Probability proportionate to size
